# Immunomodulation and Thrombolytic Approaches in the Management of Deep Vein Thrombosis and Pulmonary Embolism

**DOI:** 10.26502/fccm.92920456

**Published:** 2025-08-08

**Authors:** Angelie Pathak, Laura Roberts, Devendra K Agrawal

**Affiliations:** Department of Translational Research, College of Osteopathic Medicine of the Pacific, Western University of Health Sciences, Pomona, California 91766 USA

**Keywords:** Deep vein thrombosis, Immunomodulation, Inflammation, Leukocyte activation, Nanoparticles, Obesity, Pulmonary embolism, STING pathway, Thrombolytic therapy, Thrombosis

## Abstract

Deep vein thrombosis (DVT) and pulmonary embolism (PE) are key initiating events in the development of venous thromboembolism (VTE), a condition associated with significant morbidity, mortality, and long-term complications. While traditional therapies have focused on anticoagulation and thrombolysis, current evidence describes the pivotal role of immune pathways in the pathogenesis and progression of thrombosis. This review explores the multifaceted mechanisms underlying DVT and PE, emphasizing the contribution of inflammation, leukocyte activation, and immuno-thrombosis to thrombus formation and embolization. Key immune players such as neutrophil extracellular traps (NETs), inflammasomes, antibodies, and the STING pathway act in concert with coagulation cascades, highlighting potential targets for therapeutic modulation. We critically evaluated and discussed the efficacy and risks associated with thrombolytic agents such as alteplase, reteplase, and tenecteplase, particularly in severe or hemodynamically unstable cases. In addition, we reviewed new and innovative approaches including immune-targeted therapies and nanoparticle-based drug delivery systems, which provide the promise of more precise, safer, and cost-effective interventions. By integrating immunologic insights with evolving thrombolytic strategies, this paper supports a more tailored approach to managing DVT and PE, with the goal of reducing recurrence, minimizing complications, and enhancing long-term patient outcomes.

## Introduction

Deep vein thrombosis (DVT) is a condition where blood clots form in the deep veins, primarily in the lower extremities, which can lead to severe complications such as pulmonary embolism (PE) [1]. When a clot from DVT migrates via the venous system to the lungs, it obstructs the pulmonary arteries, causing PE [2]. DVT and PE comprise venous thromboembolism (VTE), a major cause and area of concern for cardiovascular morbidity and mortality worldwide. While anticoagulation and thrombolysis are the focus of treatment, a vast majority of patients experience recurrent events, bleeding complications, or long-term consequences of post-thrombotic syndrome and chronic thromboembolic pulmonary hypertension.

In this article we discussed the immune mechanisms involved in thrombosis and how understanding these pathways can guide the development of new therapeutic strategies to enhance thrombolytic therapy, particularly by targeting immune pathways to prevent the escalation of thrombotic events and reduce complications such as pulmonary embolism (PE). Recent research emphasizes the role of innate immunity including neutrophil extracellular traps (NETs), monocyte tissue factor expression, and complement activation in promoting thrombus initiation and propagation. This review serves to bridge the gap between thrombosis and inflammation by exploring key immune interactions, providing insights into potential immunomodulatory approaches to improve clinical outcomes.

## Epidemiology

Venous thromboembolism (VTE), which includes both deep vein thrombosis (DVT) and pulmonary embolism (PE), is a significant cause of morbidity and mortality globally, with varied incidence based on age, comorbidities, and risk exposures.

**Figure 1:**
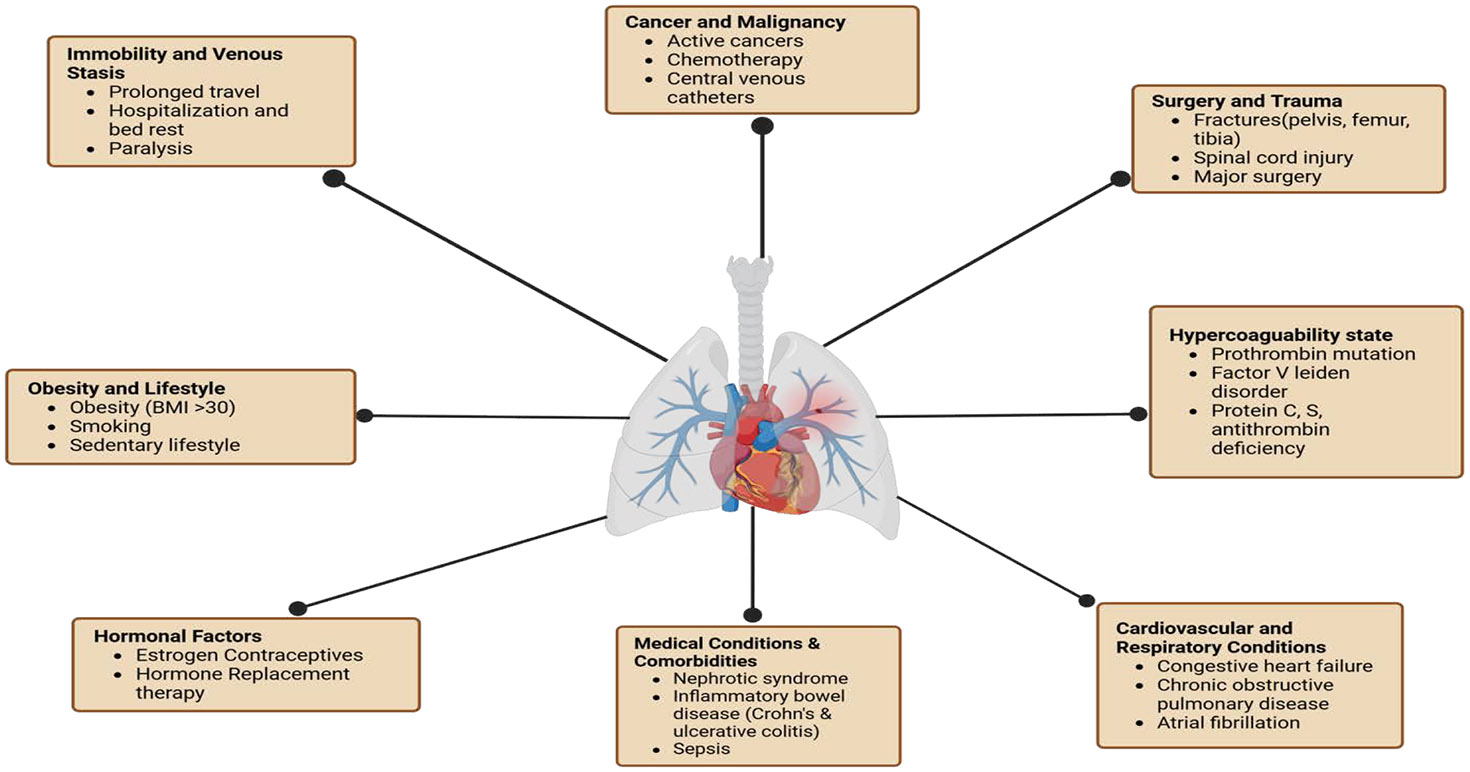
Risk factors contributing to deep vein thrombosis and pulmonary embolism. Categories of risk factors include immobility and venous stasis, cancer and malignancy, surgery and trauma, hypercoagulable states, cardiovascular and respiratory conditions, medical comorbidities, hormonal influences, and lifestyle factors such as obesity and smoking.

DVT is common among the general population, with an annual incidence of 88 to 112 cases per 100,000 person-years. A variety of factors can increase the risk, including genetics, age, surgery, hospitalizations, trauma, cancer, immobility, sleep, stress, estrogen medications, tobacco use, and infections [3-6]. These factors can trigger the development of deep vein thrombosis and pulmonary embolism (Figure 1). Within Western populations, the lifetime risk of DVT is estimated at approximately 1 in 12, whereas Asian populations tend to have the lowest incidence [7,8]. Within first-time diagnosed individuals, 25–30% have no known risk factors [9]. Additionally, recurrence is a common concern, with rates at 20–36% over ten years post first occurrence. Post-thrombotic syndrome (PTS) is a notable contributor to long-term morbidity and mortality. PTS is described as a common chronic result affecting up to 50% of people, beginning around an average of 3–6 months after the first occurrence of DVT. Prevalence of PTS is wide-ranging but is commonly associated with venous hypertension due to continual venous outflow and valve malfunctioning [10]. Symptoms of the syndrome present as changes in the tissues, capillary leaks, and chronic inflammation. These manifest as leg pain, hyperpigmentation, eczema, ulcers, pruritus, and paresthesia [11,12].

Pulmonary embolism has seen incidence rates at a slightly lower range, around 60–120 cases per 100,000 individuals, with a total average of 370,000 U.S.-based cases per year. Age over 75 is a strong correlating factor. Other risk factors such as prior VTE, thrombophilia's, immobilization, and estrogen exposure are also associated with PE. Among all VTE cases, 75% are DVT alone, whereas 25% are pulmonary embolism. Mortality is a potential outcome in those with hemodynamic instability and comorbidities, with rates ranging from 14–20% [13-15].

## Risk Factors for Deep Vein Thrombosis

### Genetic Factors in Deep Vein Thrombosis:

Genetic mutations play a significant role in the development of deep vein thrombosis (DVT), with some of the most well-established being Factor V Leiden mutation and protein C deficiency.

Factor V Leiden results from a G1691A point mutation in the F5 gene, leading to the production of a mutated factor V protein that is resistant to inactivation by activated protein C (APC). This resistance causes a hypercoagulable state, as factor Va remains active longer than normal, promoting continued thrombin generation and fibrin formation [16,17]. Individuals who are heterozygous for the mutation have a 3- to 8-fold increased risk of developing DVT, while homozygotes may have a 9- to 80-fold increased risk [18].

Protein C is a vitamin K–dependent serine protease that plays a crucial role in anticoagulation. When activated (as APC), it degrades factors Va and VIIIa with the assistance of protein S as a cofactor, thereby downregulating thrombin formation. In protein C deficiency, this anticoagulant mechanism is impaired, resulting in excessive thrombin generation and uncontrolled fibrin formation [19,20]. Two types of protein C deficiency exist: Type I is a quantitative deficiency characterized by reduced levels of circulating protein C, while Type II is a qualitative defect in which protein C levels are within normal ranges but its functional activity is impaired. Individuals with mutations in the PROC gene can present with various forms of protein C deficiency, ranging from asymptomatic cases to recurrent thrombosis [21].

The severity of protein C deficiency varies. Heterozygous individuals often experience a mildly increased thrombotic risk, while homozygous or compound heterozygous forms, particularly congenital deficiencies, can lead to severe thrombotic manifestations, including neonatal purpura fulminans a life-threatening condition marked by disseminated intravascular coagulation and skin necrosis [22].

Together, these genetic factors underscore the importance of thrombophilia screening in individuals with unprovoked DVT, strong family history, or recurrent thrombotic events, especially at a young age [23].

### Acquired Factors:

Obesity is a significant risk factor for DVT, as it contributes to chronic inflammation and increased production of pro-coagulant factors like von Willebrand factor and factor VIII. Individuals with obesity often experience decreased physical activity and increased abdominal pressure, leading to venous stasis [24,25].

Surgical procedures often result in tissue injury, which triggers a hypercoagulable state due to inflammation and endothelial damage, leading to increased levels of von Willebrand factor and fibrinogen. Cancer, particularly cancers of the pancreas, lungs, and stomach, exacerbates DVT risk by releasing tissue factor and inflammatory cytokines that activate endothelial cells, platelets, and leukocytes [26].

Age is also a significant factor, with younger individuals (<1 per 10,000 annually) having a much lower risk compared to the elderly population (1% incidence in those >60 years). Aging often correlates with the accumulation of comorbidities like obesity and inflammation, both of which promote thrombosis [27,28].

### Lifestyle Factors:

Lifestyle factors, including smoking, oral contraceptive use, poor sleep, and chronic stress, play a significant role in DVT development. Chronic stress promotes inflammation and a hypercoagulable state through upregulation of C-reactive protein and activation of the hypothalamic-pituitary-adrenal axis, leading to increased levels of coagulation factors [29]. Smoking exacerbates this by promoting oxidative stress, endothelial dysfunction, and platelet aggregation, further elevating DVT risk.

Tobacco use, including both smoking and vaping, further exacerbates thrombotic risk through multiple mechanisms. Smoking promotes systemic inflammation, leading to increased oxidative stress and downregulation of endothelial nitric oxide synthase (eNOS), which reduces nitric oxide (NO) production and impairs vasodilation, ultimately decreasing blood flow. Additionally, tobacco exposure activates monocytes and macrophages, triggering the release of reactive oxygen species (ROS). These ROS inhibit the synthesis of tetrahydrobiopterin (BH4), a critical cofactor required for proper eNOS function. The resulting BH4 deficiency further impairs NO production, compounding endothelial dysfunction and promoting a prothrombotic vascular environment [30]. Oral contraceptives can further exasperate effects, due to their high estrogen content, increasing DVT risk by promoting pro-coagulant factors like fibrinogen, prothrombin, and factors VII, VIII, IX, and X [31].

### Pathogenesis of DVT and PE:

**Figure 2:**
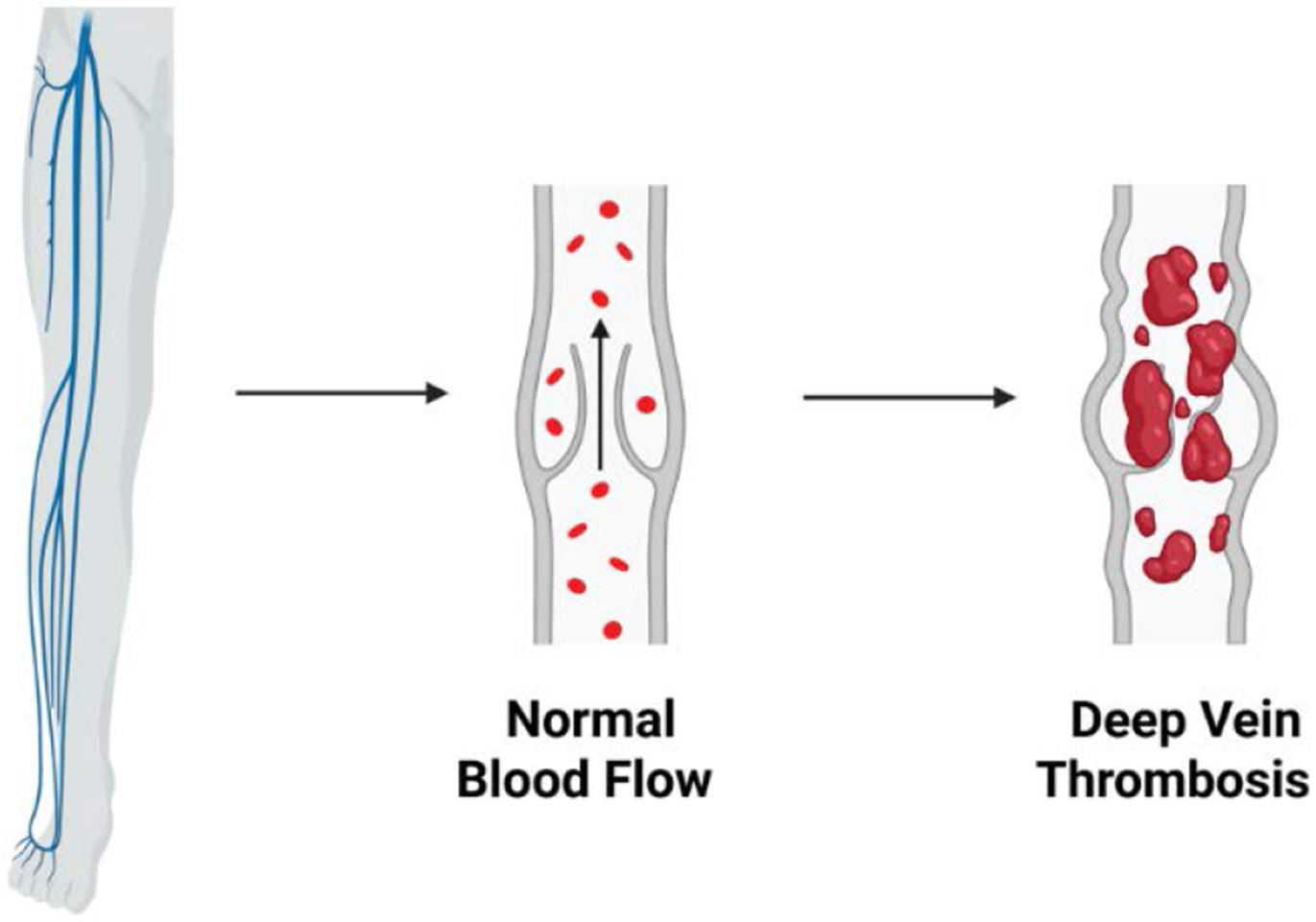
Deep vein thrombosis within a lower extremity vein, showing a thrombus obstructing normal blood flow, emphasizing the site and impact of clot formation.

Deep vein thrombosis (DVT), particularly in the lower extremities, can lead to pulmonary embolism (PE) when thrombi migrate to the pulmonary arteries and obstruct blood flow to the lungs [32]. The occurrence of PE is commonly linked to the embolization of clots from distal sites in the deep venous system (Figure 2). A retrospective study analyzing 1,585 patients with DVT found that 458 developed PE, either symptomatic or asymptomatic [33]. Another study noted a 58% prevalence of PE among individuals with DVT, with proximal acute lower extremity DVT identified as a strong predictive factor [34]

Several factors influence thrombus mobility and the likelihood of embolization, including increased blood flow, physical activity, body positioning, and structural characteristics of the clot. Thrombi with more elastic fibrin networks, increased pore size, and larger overall size are more prone to dislodgment and embolization [35]. Additionally, comorbid conditions such as heart failure, the use of central venous catheters, and hypotension are associated with an elevated risk of thrombus migration [36].

**Figure 3:**
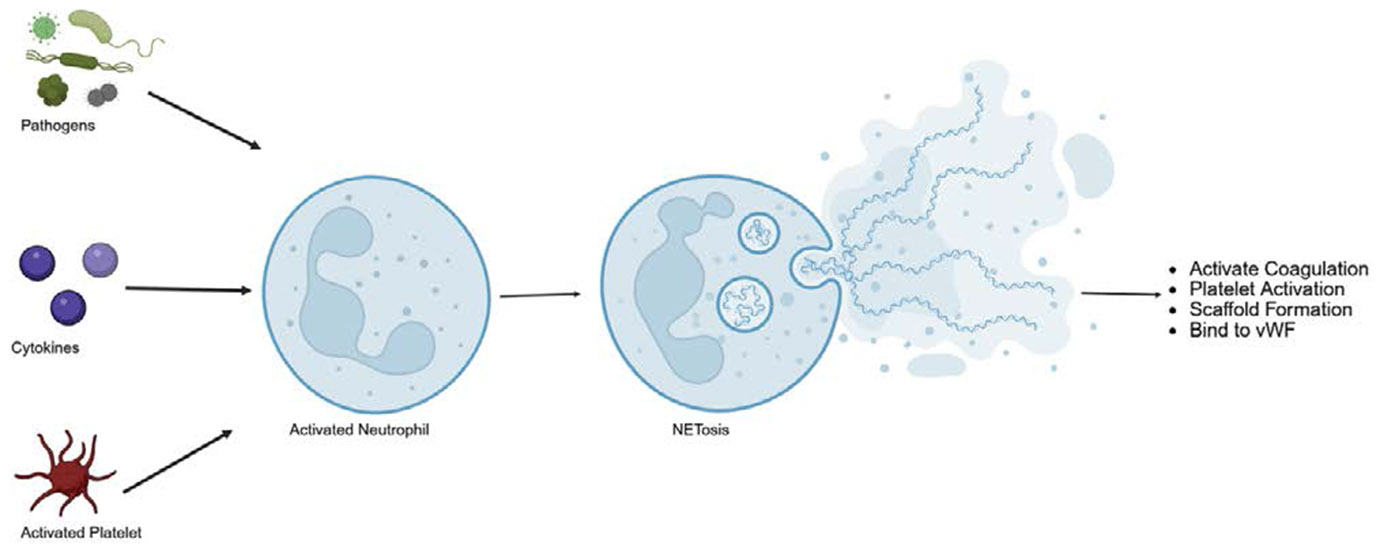
Neutrophil extracellular trap (NET) formation, or NETosis, is activated when neutrophils encounter pathogens or activated platelets, leading to chromatin de-condensation and the release of DNA, histones, and granule proteins into the extracellular space. These web-like NETs not only trap pathogens as part of the innate immune response but also promote thrombosis by activating factor XII, enhancing platelet aggregation via von Willebrand factor, and degrading TFPI to amplify both intrinsic and extrinsic coagulation pathways.

At the cellular level, the pathogenesis of DVT and PE involves not only disturbances in blood flow and coagulation but also complex immune-mediated mechanisms. Inflammation plays a critical role in initiating and propagating thrombosis. The concept of immunothrombosis describes how immune cells—particularly neutrophils, monocytes, and platelets—interact with the coagulation cascade to promote thrombus formation. Neutrophil extracellular traps (NETs) provide a scaffold for platelets and fibrin, enhancing clot stability and growth (Figure 3). In parallel, monocyte-derived tissue factor expression and activation of the complement system amplify thrombin generation and fibrin deposition, linking innate immune activation directly to clot formation [37,38].

Within the pulmonary vasculature, embolized thrombi trigger endothelial dysfunction, local cytokine release, and vasoconstriction, leading to increased vascular resistance and impaired gas exchange. These inflammatory responses not only contribute to the acute hemodynamic burden but also to long-term vascular remodeling [39].

Pulmonary embolism is the third most common cause of vascular death and is associated with significant long-term complications, including chronic thromboembolic pulmonary hypertension (CTEPH) and post-pulmonary embolism syndrome (PPES) [40]. CTEPH is characterized by persistent obstruction of the pulmonary arteries, increased pulmonary vascular resistance, and progressive right heart failure. PPES, on the other hand, involves residual thrombi that impair pulmonary gas exchange, contributing to ongoing dyspnea and reduced functional capacity [39].

The pathogenesis describes the multifactorial nature of DVT and PE, where hemodynamic, structural, and immunologic factors converge to influence thrombus formation, embolization, and long-term outcomes. Tailored approaches can therefore be best utilized to achieve targeted and effective results.

## Immune Pathways in DVT

Immune pathways play a pivotal role in the initiation and propagation of DVT. Understanding these pathways can inform novel therapeutic strategies aimed at modulating immune responses to optimize thrombolytic therapy.

## Immune Pathways and Thrombosis

**Figure 4:**
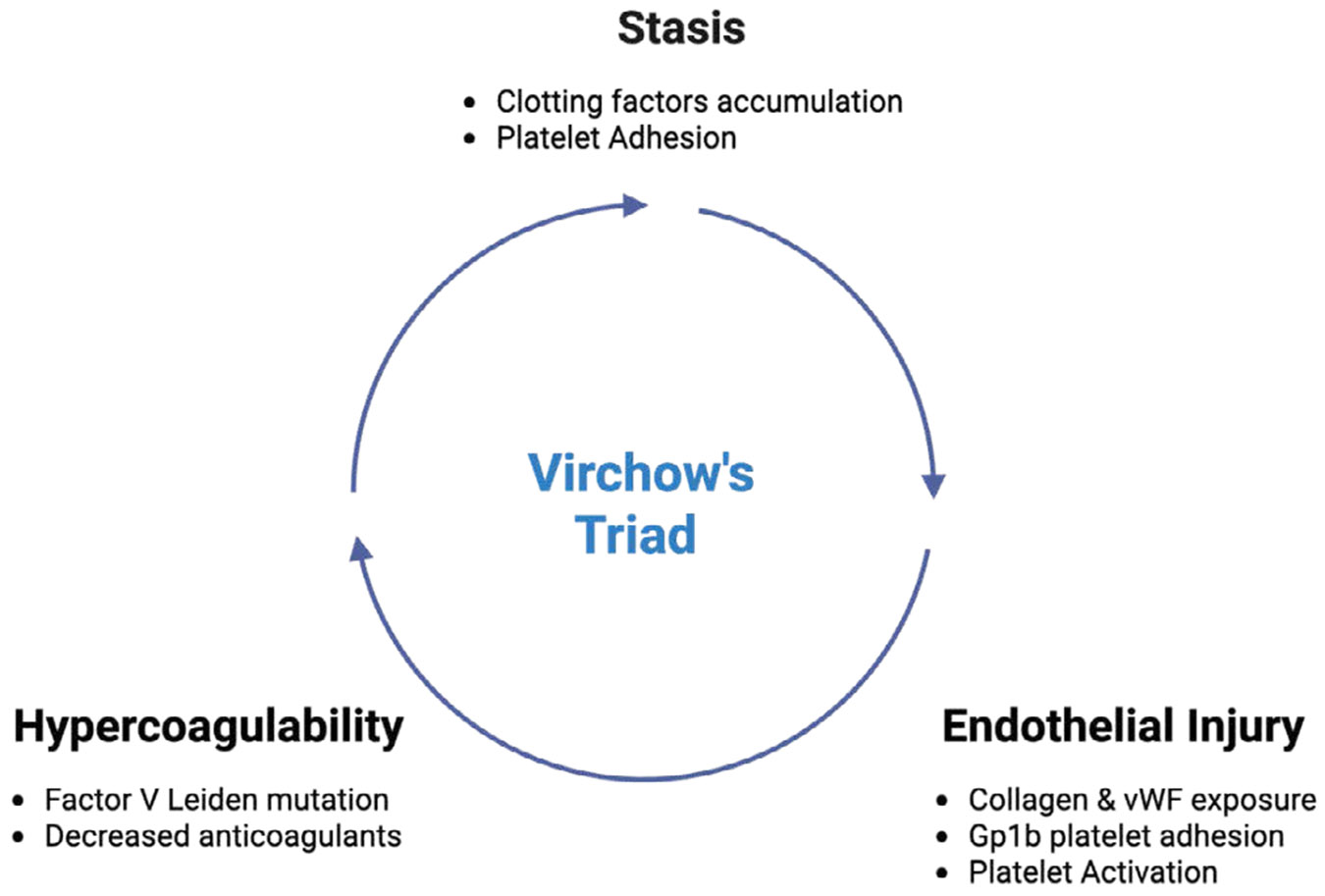
Virchow's Triad describes the three primary factors contributing to thrombosis: endothelial injury, stasis of blood flow, and hypercoagulability. This describes the interplay in the increased risk of clot formation within the vascular system.

Virchow’s Triad describes the underlying three contributing factors in the pathogenesis of thrombosis. These factors include endothelial injury, stasis of blood flow, and hypercoagulability, leading to risk of clot formation within the vascular system (Figure 4). Activation of leukocytes via thrombin and protease-activated receptor 1 (PAR-1) acts reciprocally with coagulation factors and platelets, which play regulatory roles in promoting inflammation. This inflammation, in turn, increases the recruitment of leukocytes, especially monocytes and neutrophils, to the site of the thrombus. Activation of inflammasomes and pro-inflammatory cytokines, such as IL-6 and TNF-alpha, can be induced by thrombin, fibrin, and fibrin degradation products. These cytokines further contribute to the inflammatory process, exacerbating thrombus formation [41-44].

**Figure 5:**
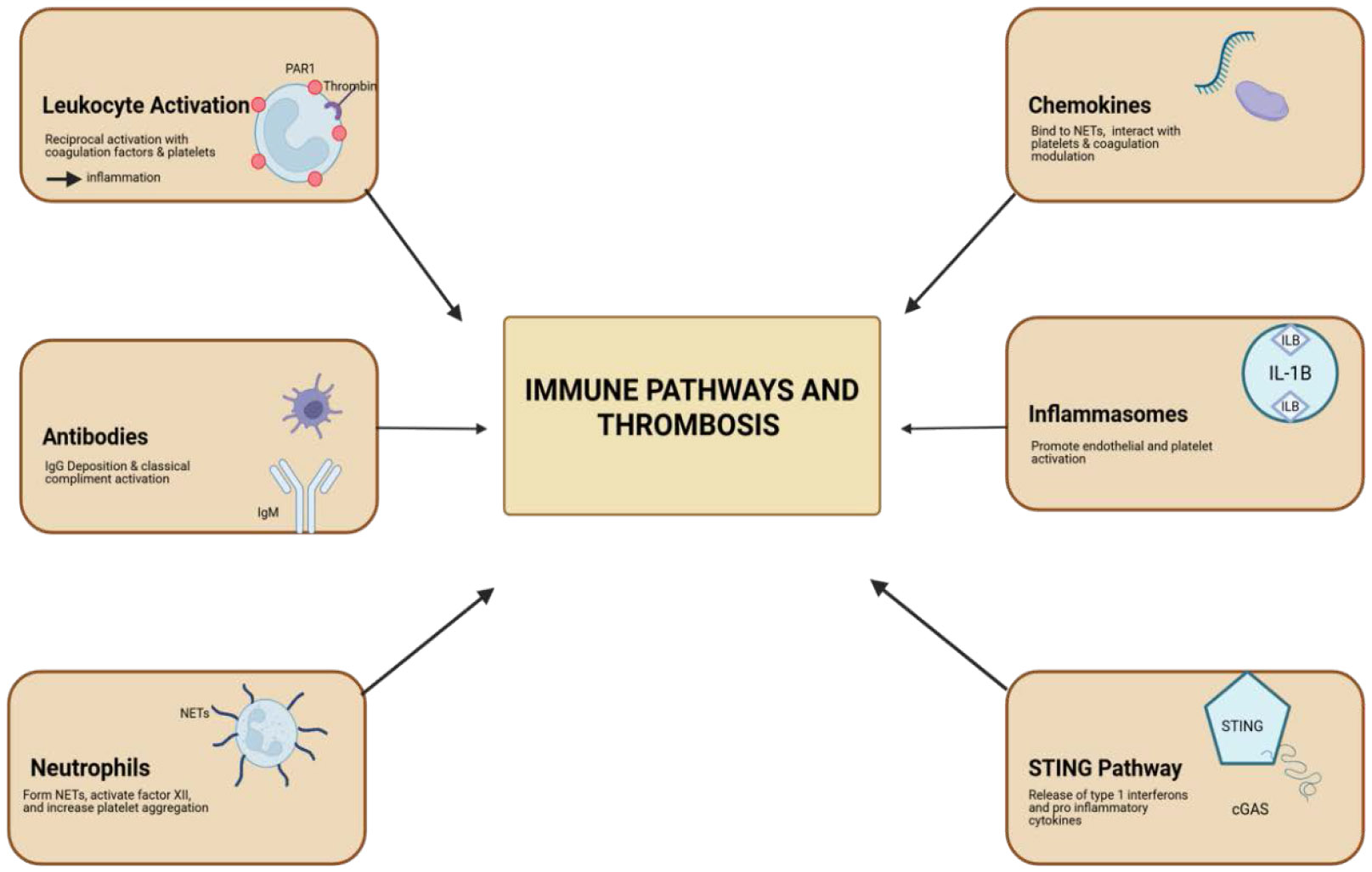
Immune pathways involved in thrombosis. Six main contributing mechanisms: leukocyte activation via thrombin and PAR-1, antibody-mediated complement activation, neutrophil extracellular trap formation, chemokine modulation of coagulation, inflammasome-driven endothelial and platelet activation, and STING pathway-mediated cytokine release.

Overall, there are six major contributing factors in the immune activation and development of thrombosis. These are shown in Figure 5 and discussed in detail in the following sections.

Chemokines and Thrombosis: Chemokines are also key players in thrombosis. They form complexes with heparin, binding to neutrophil extracellular traps (NETs), interacting with platelets, and modulating coagulation and fibrinolysis (Figure 3). Specific chemokines, particularly CXCL12, can act independently of classical functioning and promote platelet aggregation and thrombosis [45].

Inflammasomes and Thrombus Formation: Inflammasomes, particularly NLRP3, are crucial in thrombosis. When activated by hypoxia-inducible factor 1-alpha (HIF-1α) due to hypoxia and oxidative stress, NLRP3 inflammasomes produce interleukin 1 beta (IL-1β) and interleukin 18 (IL-18). These cytokines promote endothelial and platelet activation in situations of DVT, contributing to thrombus formation [46].

### Antibodies and Thrombosis:

Antibodies, particularly IgM and IgG, play critical roles in thrombus formation. Through binding of FcμR and the polymeric immunoglobulin receptor (pIgR) to platelets and endothelial cells, these antibodies enable IgG deposition that is independent of Fcγ receptor (FcγR) engagement. This unique mechanism initiates the classical complement cascade, contributing significantly to both thrombotic and inflammatory amplification [47].

This pathway is most active under conditions of reduced blood flow, where inflammation and thrombosis enter a self-sustaining feedback loop. The classical complement pathway, in this context, is activated through C1q binding, which in turn triggers the cleavage of complement proteins C3 and C5 propelling further immune-mediated thrombus growth. Notably, the complement activation occurs independently of Fcγ interaction, distinguishing it from traditional antibody-mediated immune responses and highlighting its role in immune-driven thrombogenesis [48].

### Neutrophils and Thrombosis

Neutrophils influence thrombosis through several interconnected mechanisms. They can acquire or express tissue factor (TF), enabling the release of microparticles that contribute to thrombus development. Neutrophil extracellular traps (NETs), composed of decondensed chromatin and granule proteins, further amplify coagulation by activating factor XII and promoting the intrinsic pathway [49].

Additionally, NETs interact with von Willebrand factor (vWF), enhancing platelet aggregation and reinforcing thrombus stability. NETs also have the capacity to degrade tissue factor pathway inhibitor (TFPI), thereby increasing TF activity and accelerating extrinsic coagulation. Through these combined mechanisms, neutrophils create a prothrombotic environment that supports both clot formation and propagation in the microvasculature and larger vessels such as the carotid artery [50].

### STING Pathway and Thrombosis

The STING pathway, also known as the stimulator of interferon genes, is a key component of the innate immune signaling response. STING is a protein located at the junction of the endoplasmic reticulum and becomes activated specifically in response to bacterial and viral infections. In mammalian cells, this activation occurs through cytosolic DNA sensing via cGAS, which produces the secondary messenger cGAMP synthase.

Once activated, STING initiates a downstream cascade involving TANK-binding kinase 1 (TBK1) and NF-κB signaling pathways. This leads to the release of type I interferons and pro-inflammatory cytokines, promoting inflammation. These transcription factors not only drive immune activation but also contribute to coagulation and thrombosis, especially in the setting of infection-triggered immune responses [51].

### Thrombolytic Therapy in Venous Thrombosis and Pulmonary Embolism

It is important to consider the risks associated with the administration of thrombolytic therapy. Bleeding risk is a significant factor when using this therapy, as studies indicate an increased overall risk of bleeding with a relative risk (RR) of 1.89 and an increased risk of intracranial hemorrhage (RR 3.17). Other studies support these findings; for example, one study comparing anticoagulant therapy to thrombolytic therapy agents showed a 2.2% bleeding rate with anticoagulants versus 6.7% with thrombolytics. Understanding the risks associated with bleeding is a critical consideration when determining if the long-term potential for negation of post-thrombotic syndrome (PTS) outweighs the risks associated with bleeding [52-54].

Additionally, some absolute contraindications according to current guidelines from the American College of Chest Physicians (ACCP) for the administration of thrombolytic therapy include active bleeding, prior intracranial hemorrhage, brain or spinal cord injury or surgery, trauma or fracture to the head, and stroke within the prior 3 months [55].

### Therapeutic Strategies Targeting Immune Pathways in DVT

Several therapeutic strategies targeting immune pathways are being explored to complement thrombolytic therapy and improve DVT treatment outcomes.

### Patient Selection Criteria for Thrombolytic Therapy

In patients with DVT, thrombolytic therapy is most used in cases of extensive iliac or femoral DVT, especially when symptoms have been present for less than 14 days. Other factors include the patient’s predictability of surviving at least one year, a low chance of bleeding, and good functional status [55]. The guidelines formed by the American College of Chest Physicians are focused on reducing the chance of developing PTS, a common long-term consequence of DVT. A study by Broderick et al. found that thrombolysis is an effective method for lysing a clot and can reduce the overall chance of developing PTS [56]. PTS is a long-term outcome of DVT, characterized by venous insufficiency, swelling, cramping, and pain at the affected site, and can manifest as eczema, varicose veins, edema, and ulcers in the leg. Symptoms may worsen during movement, such as walking or running [57]. The Villalta scale is used to identify the stage of PTS, with scores ranging from mild (5-9), moderate (10-14), and severe (15+) [58].

### Thrombolytic Therapy in Pulmonary Embolism

In patients experiencing PE, thrombolytic therapy is recommended for cases with hemodynamic instability, such as those with acute PE or hypotension (<90 mmHg). In these cases, thrombolysis is considered the primary course of action due to its ability to improve the function of the right ventricle and reduce the pressure on the pulmonary arteries by breaking down the thrombus [55].

Common thrombolytic agents used include alteplase, reteplase, and tenecteplase. These are often used to target clots through tissue plasminogen activators. Alteplase is administered intravenously over the course of two hours. It works by binding to the fibrin in a thrombus, allowing for fibrinolysis and clot breakdown by converting plasminogen to plasmin. This helps relieve pressure within the pulmonary artery, increases the functioning of the right ventricle, and maintains the patency of the pulmonary arteries, promoting normal circulation [55]. However, alteplase is associated with an increased risk of bleeding, with the potential for intracranial hemorrhage ranging between 2-5%, and less severe bleeding being more common in about 68% of patients [59].

Reteplase functions similarly to alteplase but differs in that it has a shorter infusion time of about one hour. It is structured as a "kringle" and protease domains and acts as a plasminogen activator, though it has a longer half-life compared to alteplase [60]. However, its affinity to bind tightly to fibrin is less, which results in a slower rate of action compared to alteplase.

Tenecteplase, acting similarly to alteplase, varies in its ability to provide specificity to fibrin molecules, decreasing overall systemic activation [61,62]. It acts more rapidly, with an intravenous dose administered over about five seconds, depending on the patient’s weight [63]. Tenecteplase is considered highly efficient, especially when a rapid thrombolytic effect is required.

Both alteplase, reteplase, and tenecteplase offer effective therapeutic measures, with similar mechanisms of action. The choice of agent depends on the specific needs and circumstances of the individual patient.

## Financial Factors

There are substantial costs associated with DVT and PE, making the financial burden on patients a significant consideration when adjusting for treatment. A retrospective study examining 28,953 and 35,550 patients, respectively, found that the average cost for DVT treatment was approximately $30,000 for an average hospital stay of 4.7 days. Pulmonary embolism (PE) alone incurred costs around $37,000 for an average stay of 5.1 days [64,65]. The highest costs were typically incurred during the first few days of hospitalization. Additionally, costs associated with complications, such as post-thrombotic syndrome (PTS), further increase baseline treatment costs by around $20,000. Furthermore, the treatment costs for DVT and PE also increase due to the use of therapy [66].

Thrombolytic therapy can contribute to these costs, but in some instances, it may be considered cost-effective as it can reduce long-term treatment needs. However, the associated costs can increase due to equipment, complication risks, and ICU care during administration. For example, the cost of alteplase is approximately $488 per treatment, reteplase costs around $1,787 per treatment, and tenecteplase falls somewhere in the middle range. These costs do not account for additional expenses related to the length of the hospital stay [67].

Therefore, understanding the patient's profile and the therapy's potential effectiveness based on these considerations is crucial when evaluating the cost-benefit analysis of thrombolytic therapy for improved patient outcomes.

## Current Treatments

A variety of ongoing developments in treatment aim to enhance the use of thrombolytics by targeting key aspects of immune functioning. Key targets include anti-inflammatory agents, neutro specialized pro-resolving mediators, cytokine modulation, and T-cell modulation.

Anti-inflammatory agents act to inhibit endothelial activation by decreasing leukocyte adhesion through reduced levels of P-selectin and E-selectin, thereby suppressing overall thrombus formation. They further act on NETs, as agents like resolvin D4 inhibit the production of NETs and eliminate scaffolds for NET formation, thereby promoting apoptosis and clearance by macrophages. Anti-inflammatory agents such as resveratrol inhibit the DVT-induced inflammatory response by suppressing the HIF-1α/NLRP3 pathway, leading to decreased expression of IL-1β, caspase-1, and tissue factor [42-44]. Blocking the NLRP3 inflammasome with inhibitors like MCC950 has been shown to reduce the weight-to-length ratio of thrombi and downregulate inflammatory factors such as IL-1β [68-70].

Additionally, targeting the STING pathway is an emerging mechanism in DVT prevention. Peptides like CST5 prevent the binding of STING to STXBP2, thereby directly inhibiting granule secretion and platelet activation in mouse models [71]. Small molecule inhibitors, such as BB-Cl-amidine, also act to inhibit STING activation, thereby reducing pro-inflammatory responses. Other compounds like SN-011 bind to the cyclic dinucleotide (CDN) pocket within STING, resulting in the inactivation of inflammatory cytokines and interferons [72].

Further inhibition of the CXCL12–CXCR4 axis using agents such as plerixafor blocks this pathway by antagonizing CXCR4, thereby reducing downstream platelet activation. Similarly, inhibition of the CCL2–CCR2 axis by RS504393 blocks CCR2 binding and the recruitment of monocytes to the thrombus site [73,74]

Monoclonal antibodies also play a role in targeting IL-1β, with agents such as canakinumab shown to inhibit endothelial activation. Anti–P-selectin drugs prevent the interaction between P-selectin and its ligands, reducing leukocyte recruitment to the site and overall thrombus formation [75,76].

## Nanoparticles

**Figure 6:**
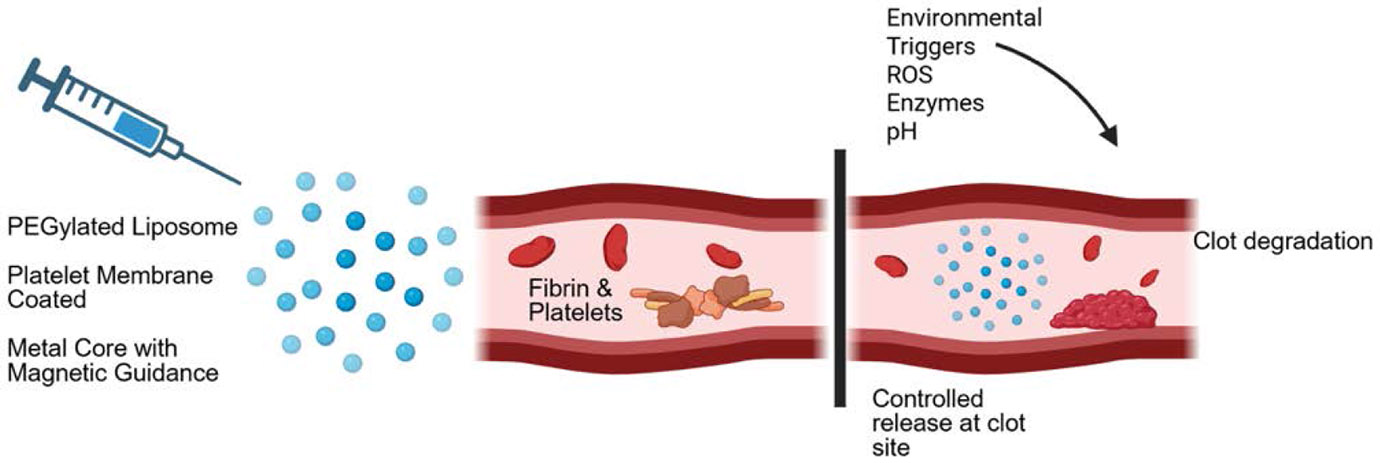
Nanoparticles are administered via injection and travel through the bloodstream. Thrombi composed of fibrin and platelets are targeted, and when the clot site is reached, the nanoparticles release their therapeutic agents, promoting clot dissolution and allowing normal blood flow. This targeted delivery enhances drug localization, minimizes systemic side effects, and improves treatment efficacy for thrombotic conditions such as deep vein thrombosis.

Nanoparticles are classified as either nanospheres or nanocapsules. Nanospheres contain the therapeutic agent dispersed within the polymer matrix, whereas nanocapsules encapsulate the agent within a polymer membrane, offering a more targeted delivery system [77]. These nanoparticles—commonly lipid-, polymer-, or metal-based—range from 1 to 1000 nanometers and enable enhanced delivery of thrombolytic agents. The nanoparticles are administered via intravenous injection and the particles travel through the blood stream (Figure 6). Their efficacy stems from their ability to prevent rapid drug inactivation, deliver therapeutics directly to the site of thrombosis, and extend the half-life of active agents, thereby ensuring stable and controlled drug release [78].

One of the most significant advantages of nanoparticles is their capacity to be functionalized with ligands or specific antibodies, allowing for precise, site-specific delivery to thrombi. Additionally, nanoparticles can be engineered to release their therapeutic payload in response to environmental stimuli such as changes in pH, enzyme activity, or physical stress. Metal-based nanoparticles, for instance, can be directed to the site of action using external magnetic forces. Alternatively, nanoparticles coated with platelet membranes can mimic the natural properties of platelets. These coatings, which contain P-selectin, glycoprotein Ib (GPIb), and integrins, enable the particles to adhere to vascular injury sites and facilitate localized drug release [79].

Several nanoparticle-based drug formulations, originally designed for oncology, have been FDA-approved and are now being explored for use in DVT treatment. For example, PEGylated liposomal doxorubicin (Doxil) and albumin-bound paclitaxel (Abraxane) are two such agents. Doxil provides a targeted delivery of doxorubicin with reduced systemic toxicity, while Abraxane minimizes hypersensitivity reactions commonly associated with solvent-based paclitaxel by using an albumin carrier [80].

Although still in the early stages, emerging research has shown promising results. A study by Cheng et al. demonstrated that multifunctional nanoparticles made from bioactive amphiphiles significantly reduced DVT in pregnant rat models. These nanoparticles not only dissolved existing thrombi and restored vessel patency but also helped prevent future thromboembolic events [81]. This was further supported by research on the AMSNP@PM-rH/A nanoplatform, which incorporates a platelet membrane coating. This coating enhanced stability, reduced accumulation in the kidneys and liver, and enabled a more targeted therapeutic approach [82].

Nanoparticles represent a novel and promising strategy for the effective delivery of thrombolytic agents, with the potential to minimize widespread systemic effects and improve patient outcomes.

## Future directions

Future research in thrombolytic therapy for DVT and PE elimination and inhibition should focus on more targeted approaches. This could involve creating therapeutics that are specifically directed toward fibrin without damaging surrounding tissues, thus minimizing potential adverse effects. One promising direction is the engineering of recombinant tissue plasminogen activator (rtPA) to enhance its fibrin-specific binding properties.

Additionally, investigating the genetic profile of individuals experiencing DVT or PE could help determine how a particular therapy will affect them. This could be achieved using genetic markers and genome-wide association studies (GWAS), which would enable clinicians to assess bleeding risks and the effectiveness of thrombus elimination on an individual basis.

Furthermore, advanced imaging techniques to monitor the clot in real time, such as 3D imaging and photoacoustic imaging, could provide the ability to adjust and fine-tune thrombolytic therapy based on the ongoing progress of clot resolution. This could reduce the need for trial-and-error approaches, improving overall patient outcomes.

Investing in innovative methods within thrombolytic therapy could provide a way to reduce complication risks, lower treatment costs, and minimize the excessive bleeding typically associated with thrombolytic therapy. These advancements would offer a more personalized, precise, and safer approach to managing DVT and PE in the future.

## Key Points

- Immune activation plays a pivotal role in DVT through thrombin-induced leukocyte activation via PAR-1, promoting inflammation and thrombus formation. Inflammasomes like NLRP3, activated by HIF-1α under hypoxia, produce IL-1β and IL-18, which trigger endothelial and platelet activation.
- Genetic and acquired factors such as Factor V Leiden and protein C deficiency, obesity, cancer, surgery, smoking, and oral contraceptive use promote venous stasis, inflammation, and endothelial dysfunction.
- Thrombolytic therapy is effective for clot lysis and reducing post-thrombotic syndrome but carries a high bleeding risk. Its use is restricted to severe cases like extensive iliofemoral DVT or hemodynamically unstable PE.
- Emerging therapies target immune mechanisms such as the NLRP3 inflammasome, STING pathway, and chemokine axes to reduce thrombus formation and inflammation. Monoclonal antibodies and anti–P-selectin agents also work to prevent leukocyte recruitment and endothelial activation.
- Nanoparticles, including platelet membrane-coated systems, allow for delivery of thrombolytics and immune modulators to clot sites, minimizing systemic side effects through engineering stimuli like pH or enzyme activity, increasing drug stability and targeted therapeutic
